# Amoxicillin/Clavulanic Acid-Induced Symmetric Drug-Related Intertrigious and Flexural Exanthema

**DOI:** 10.7759/cureus.33849

**Published:** 2023-01-16

**Authors:** Gulistan Alpagat, Ayse Baccioglu, Betul Dumanoglu, Sumeyra Alan Yalim, Ayse Fusun Kalpaklioglu

**Affiliations:** 1 Department of Immunology and Allergic Diseases, Kirikkale University Faculty of Medicine, Kirikkale, TUR

**Keywords:** anti-histamin, steroid treatment, amoxicilline, sdrife, drug-induced hypersensitivity

## Abstract

β-Lactams, particularly penicillins, may cause several allergic reactions. We described symmetric drug-related intertriginous and flexural exanthema (SDRIFE) illness in this case, a rare instance of systemic contact dermatitis caused by amoxicillin/clavulanic acid that needs to be considered in the differential diagnosis. A 65-year-old male patient was admitted to our Allergy Outpatient Clinic because of increased blue-purple pigmentation on the flexural surfaces of the hip, forearm, axilla, and posterior face of the neck. The patient was receiving a combination of angiotensin receptor blocker (ARB) and hydrochlorothiazide diuretic medication for hypertension. The patient used an antibiotic containing amoxicillin three months ago; As a result, there was localized redness, itching, and black spotting without any systemic symptoms. Similarly, the patient reported that when he used amoxicillin for an upper respiratory tract infection eight months ago, he experienced similar side effects within 20 days and recovered when he applied corticosteroid ointment.

Due to the symmetrical site involvement following the consumption of penicillin group antibiotics with a five-month gap and subsequent comparable reactions in our patient, SDRIFE was taken into consideration. The results of the skin punch biopsy identified Baboon Syndrome (SDRIFE). Treatment with topical corticosteroids and antihistamines began. Clinically speaking, SDRIFE is distinguished by significant erythema of the gluteal/perianal area and/or V-shaped erythema of the inguinal/perigenital area, symmetric involvement of at least one other intertriginous or flexural area, and the absence of systemic signs or symptoms. The possibility that the medication may have contributed to the patient's erythematous eruption in the flexural regions should be taken into account, and the patient should be advised to stop taking the medication and not use it again.

## Introduction

Different allergic reactions that are categorized as early or immediate and delayed type can be brought on by β -lactams. The most frequent allergic reactions to penicillin are maculopapular eruptions and urticaria. Even though maculopapular eruptions are the most common late-type reactions, there are some rare ones such as symmetrical drug-related intertriginous and flexural exanthema (SDRIFE). After exposure to systemic medications, this reaction manifests as a symmetrical erythematous rash on the gluteal and intertriginous areas. Since the location of the lesions on the buttocks and inner thighs mimics the red rump of baboons, SDRIFE is also known as "baboon syndrome"[1]. Regardless of antecedent sensitization, Hausermann et al. advocated the name SDRIFE as more suitable for reactions that happen following exposure to systemic medicines or local injection of contact allergens and pharmaceuticals [2]. Ceftriaxone, penicillin, and erythromycin were listed as the most frequent causes of SDRIFE [3]. Here, we describe a rare instance of SDRIFE who, upon treatment with amoxicillin and clavulanic acid, experienced a delayed reaction.

## Case presentation

A 65-year-old man was referred to our Allergy Outpatient Clinic in with a rash that sprang out of nowhere, itching and burning in the flexural areas of the extremities, then becoming blue-purple. No overall symptoms existed. For five days, he took amoxicillin-clavulanic acid (875mg/125mg, twice a day) po with symptoms of upper airway infection. Although his symptoms subsided, 4-5 days after stopping the medicine, skin issues appeared. Eight months ago, he had experienced similar skin manifestations in a milder delayed reaction to the same drug. Since there was no definitive diagnosis at that time, the reaction was not thought to be drug-related. Skin lesions were partially resolved with topical corticosteroids, and brown spots remained. He had no previous history of contact with allergen administration.

**Figure 1:**
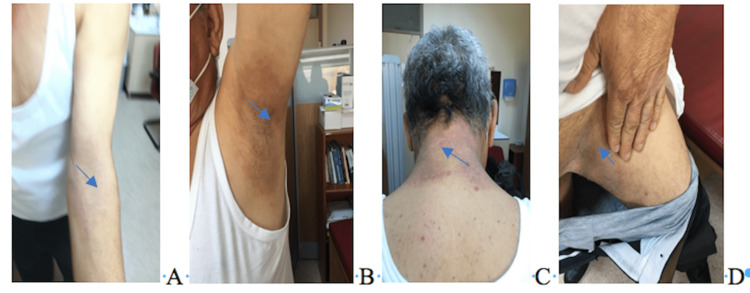
Skin exanthema of various sites due to SDRIFE (A: Flexural forearm, B: Axillar area, C: Posterior neck, C: Inguinal region).

Dermatological examination of the posterior neck and the intertriginous area revealed many bilateral, symmetrical, well-demarcated blue-purple macules and plaques involving the axillary, flexures of the extremities, inguinal, and gluteal regions (Figure 1). In the form of pustules, papules, purpura or bullae. There were no skin lesions on the palms, soles, or mucosae. A systemic analysis turned out nothing unusual. Urinalysis results and blood cell counts were both normal. For a superficial fungal infection, direct microscopic inspection and culture produced negative results.

**Figure 2:**
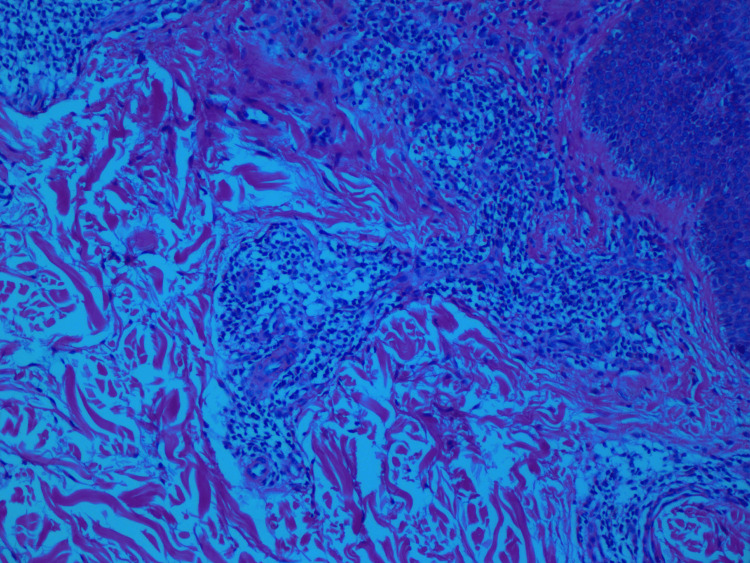
Perivascular inflammation

**Figure 3:**
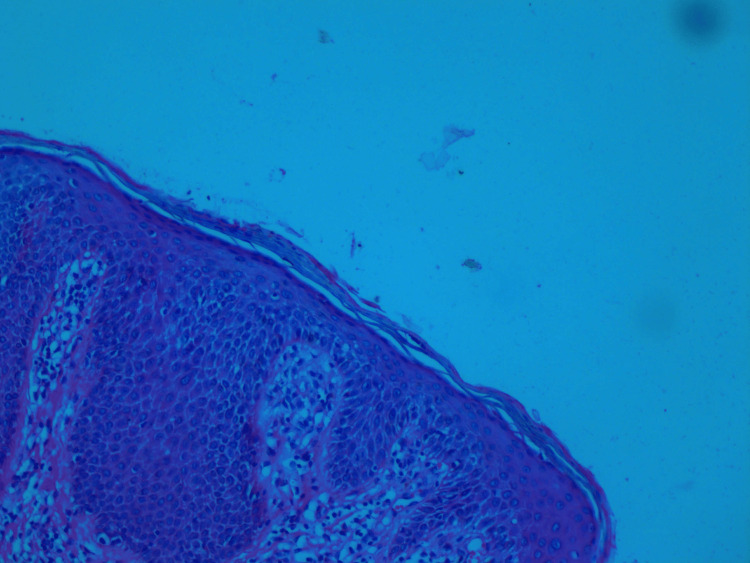
Parakeratozis and spongiosis

Skin biopsy from the inguinal region was histopathologically examined, and the results were consistent with SDRIFE. They revealed focal parakeratosis of the epidermis, significant spongiosis, irregular acanthosis, and perivascular moderately mixed inflammation in the dermis (Figure 2). Based on histology and clinical characteristics, SDRIFE was defined (Figures 2, 3).

Based on histology and clinical characteristics, SDRIFE was defined. The medication was dissolved in distilled water and petrolatum with a 30% concentration before being used for the patch test [4]. After that, prick and intradermal tests with penicillin (DAP penicillin® (Diater laboratories, Madrid, Spain), penicillin G, and ampicillin/amoxicillin preparations) were performed and yielded negative. For prick testing, amoxicillin/clavulanic acid in tablet forms was mashed and diluted with 1 mL physiological saline solution. An intradermal test was not performed because of parenteral amoxicillin-clavulanic acid form is unavailable in Turkey. Although the gold standard test for drug allergy was a drug provocation test with the offending drug, this was not done because the patient had a broad reaction that had been getting worse over time [5]. With prednisolone (40 mg/day) for five days and p.o. anti-histamine for 20 days, his skin lesions significantly improved. It is recommended to avoid medications containing amoxicillin/clavulanic acid.

## Discussion

Here, we described an uncommon disorder called SDRIFE, a unique variation of eczematous skin diseases with a small number of documented instances. We hypothesize that the real prevalence of SDRIFE is likely higher than previously believed. According to clinical criteria, SDRIFE can be defined by the development of symmetrical, sharply delineated erythema on the buttocks and/or thighs after exposure to systemic medications, involvement of the flexural fold, and the absence of systemic symptoms [6]. With axillary regions localized, symmetrical eruptions, and no systemic symptoms, this patient satisfied all the criteria. Interestingly, despite the disease's name being derived from this participation, our patient's buttock regions were not affected. Although the cause of the lesions' usual placement in SDRIFE is unknown, it has been proposed that inflammation and blockage of sweating in these areas may be to blame [7]. When there has been prior sensitization, the site of the lesion is comparable to that of allergic contact dermatitis; however, when there has not been prior sensitization, the lesion takes the appearance of extensive eczematous plaques that have the propensity to coalesce [2].

Fixed drug eruption, acute generalized exanthematous pustulosis, and drug rash with eosinophilia and systemic symptoms are among the differential diagnoses. Lesions on the face and palmoplantar surfaces are not prevalent in SDRIFE, and neither are mucosal or systemic involvements at the time of presentation [8].

As in this case, SDRIFE lesions have maculopapular erythema with itching. Pustules, papules, blisters, purpuric lesions, and involvement of the palms, soles, face, or mucosae are uncommon atypical manifestations [8]. Given that our case was a man, which is consistent with the research, it is more common in men [9].

Since the pathophysiology can mirror acute generalized pustular dermatosis, bullous drug eruptions, lichenoid dermatitis, vasculitis, or dermatoses, the clinical diagnostic criteria are crucial. The reaction, which resembles a type IV delayed-type hypersensitivity immune reaction, typically manifests 1 to 14 days after the medicine was taken [2,10]. The most often reported drugs in SDRIFE include chemotherapeutic agents, radio-contrast agents, non-steroidal anti-inflammatory drugs, anti-hypertensives, and anti-infective agents, particularly β -lactam antibiotics [2,9,11]. Drug challenge tests are not required [12]; however, they can be useful in identifying the offending substance. In our case, skin testing was unable to detect the culprit drug. However, a previous history of a similar reaction and the recurrence of the same skin lesions following the patient's second dose of amoxicillin/clavulanic acid can be regarded as signs of an unintentionally positive drug provocation test. Treatment for SDRIFE involves stopping the culprit drug and then using topical or systemic corticosteroids. After receiving treatment with oral corticosteroid (40 mg for 10 days) and antihistamine, as well as topical corticosteroid on the afflicted areas, our patient showed improvement.

## Conclusions

It is crucial to mention this unusual drug reaction because, unless the connection between the drug intake and the condition is not noted, it can be easily missed. This case was intriguing because it had an unusual diagnosis and no buttock skin eruptions. In situations of symmetrical intertriginous involvement and flexural exanthema, a thorough history must be collected due to the common usage of amoxicillin/clavulanic acid. Based on histology and clinical characteristics, SDRIFE was diagnosed, and also other diagnoses were excluded with a skin biopsy.
